# Conidia of the insect pathogenic fungus, *Metarhizium anisopliae*, fail to adhere to mosquito larval cuticle

**DOI:** 10.1098/rsos.140193

**Published:** 2014-10-22

**Authors:** Bethany P. J. Greenfield, Alex M. Lord, Ed Dudley, Tariq M. Butt

**Affiliations:** 1 College of Science, Swansea University, Singleton Park, Swansea SA2 8PP, UK; 2 College of Engineering, Swansea University, Singleton Park, Swansea SA2 8PP, UK; 3 College of Medicine, Swansea University, Singleton Park, Swansea SA2 8PP, UK

**Keywords:** atomic force microscopy, *Metarhizium*, mosquito larvae, lipidomics

## Abstract

Adhesion of conidia of the insect pathogenic fungus, *Metarhizium* *anisopliae*, to the arthropod host cuticle initially involves hydrophobic forces followed by consolidation facilitated by the action of extracellular enzymes and secretion of mucilage. Gene expression analysis and atomic force microscopy were used to directly quantify recognition and adhesion between single conidia of *M. anisopliae* and the cuticle of the aquatic larval stage of *Aedes aegypti* and a representative terrestrial host, *Tenebrio molitor*. Gene expression data indicated recognition by the pathogen of both hosts; however, the forces for adhesion to the mosquito were approximately five times lower than those observed for *Tenebrio*. Although weak forces were recorded in response to *Aedes*, *Metarhizium* was unable to consolidate firm attachment. An analysis of the cuticular composition revealed an absence of long-chain hydrocarbons in *Aedes* larvae which are thought to be required for fungal development on host cuticle. This study provides, to our knowledge, the first evidence that *Metarhizium* does not form firm attachment to *Ae. aegypti* larvae *in situ*, therefore preventing the normal route of invasion and pathogenesis from occuring.

## 2. Introduction

*Metarhizium anisopliae* is a widespread, soil-borne fungal pathogen of insects, ticks and mites [1–3] with much potential as an environmentally friendly alternative to conventional chemical pesticides for the control of pests of socio-economic importance [3]. *Metarhizium* infection processes are similar to those of other entomopathogenic fungi; they entail conidial attachment to the host surface followed by germination and penetration of the host cuticle through a combination of enzymatic activity and mechanical force [4–6]. The fungal cell wall is critical in the infection process and is involved in numerous essential functions, including protection, osmotic stability, morphogenesis and cell–cell interactions, specifically host recognition and adhesion. It mainly comprises many proteins associated with host–pathogen interactions located in the outer cell wall bound covalently to *β*-1,6-glucans. These proteins include the superoxide dismutases, phospholipases, aspartyl proteases and adhesins [7,8]. Molecular recognition through these proteins is a key event in pathogenesis, with the initiation of the infection process occuring between fungal adhesins and specific receptors in the host cuticle [9].

From the hosts perspective, the arthropod cuticle is the primary and possibly most important barrier to the external environment and in particular fungal pathogens [10,11]. Its surface structure, topography and chemical composition can influence spore adhesion and therefore fungal pathogenicity [11,12], with hydrophobic lipids and fungistatic compounds within the outermost epicuticular wax layer playing an essential role in attachment and germination of fungal propagules [11,13–15]. Firm adhesion of conidia is crucial for the success of the pathogen as this can influence specificity and virulence.

Conidial attachment is a two-step process. The first step involves passive attachment mediated through a combination of non-specific hydrophobic and electrostatic forces as well as attachment via specific ligands or adhesin protein interactions [16,17]. Hydrophobins, in particular, found in the outer layer of the spore cell wall, mediate adhesion to hydrophobic components of the arthropod cuticle [16,18–20]. The second step involves secretion of enzymes to create an infection court. Hydrolytic enzymes degrade fungistatic fatty acids, release nutrients and facilitate penetration of the cuticle [21–25]. Mucilage is often secreted to consolidate the attachment of the fungus to the host surface. The adhesins, *Mad1* and *Mad2*, assist in attachment of the fungus to insect and plant surfaces, as a result *Mad1* also contributes to pathogenesis [17,26].

Recently, strains of *Metarhizium* were reported to be highly pathogenic to mosquito larvae [27,28], with death following the ingestion of conidia as opposed to adhesion and penetration of the cuticle [29]. Exactly why the conidia did not adhere is unclear. This study therefore sought to study the attachment process of an individual spore to host cuticle for both the mosquito larvae and a representative terrestrial invertebrate host. The attachment process for both was studied in the mosquito's usual aqueous environment in order to determine whether non-specific factors such as water interferes with the attachment process, thereby allowing the investigation of fungus-host-specific determinants of successful or unsuccessful attachment. Typically, spore adhesion is measured using count methods, however, recent advances in atomic force microscopy (AFM) have enabled the direct measurement of the adhesion forces of a single particle or cell and their interacting surfaces [30]. This study combines the use of AFM, gene expression and lipidomic analyses to further investigate the host–pathogen interactions of *Metarhizium* to its insect host. The expression of the *Metarhizium* adhesin genes (*Mad1* and *Mad2*) in response to terrestrial insect cuticle and aquatic mosquito larvae was used as an indicator of the recognition of the cuticle as a cue for pathogenesis to be initiated by the fungus. The AFM measurements were used to determine whether this cue was allowing traditional infection processes to be successful while the lipidomic analysis studied the two cuticle ‘trigger’ sources from a chemical perspective. The integrated data allow for a more detailed study of the processes and mechanisms that influence the infection process in aquatic mosquitoes compared with the more commonplace, terrestrial insects targeted by the fungus.

## 3. Material and methods

### 3.1 Samples, RNA extraction and cDNA synthesis

Ten *Aedes aegypti* larvae per replicate were either inoculated with 50 ml of 1×10^7^ conidia ml^−1^ solution of *M. anisopliae* ARSEF 4556 or with distilled water and incubated at room temperature for 24 h. After 24 h, the mosquitoes were harvested, washed in distilled water and frozen under liquid nitrogen. In addition, three *Tenebrio molitor* larvae, per replicate were subjected to an immersion assay in 50 ml of 1×10^7^ conidia ml^−1^ ARSEF 4556 for 20 s, the insects were then incubated on moist filter paper at 27°C for 24 h, before freezing under liquid nitrogen. All samples were prepared in triplicate.

Samples were ground with a micropestle and total RNA extractions carried out using the RNeasy Micro kit (Qiagen) following the manufacturer's instructions. RNA concentration and purity was assessed at 260 and 280 nm absorbance using a Nanodrop 2000 (Thermoscientific). Total RNA (1 μg) was reverse transcribed using the QuantiTect Reverse Transcription kit (Qiagen) with gDNA elimination reaction, for the experiment to quantify fungus-derived transcripts.

### 3.2 qPCR assay for expression of adhesin genes

Relative cDNA quantity was analysed by using the qPCR method previously described [29]. The same proctocol was followed, using 18S rRNA and elongation factor tEF as reference genes. Transcript levels were determined using the CFX96 Real-Time PCR detection system (Biorad). PCR reactions were performed in 10 μl volumes consisting of 1 μM of each primer, 2 μl of cDNA sample, 5 μl SYBR Green Fastmix (Quanta) and 1 μl diethylpyrocarbonate-treated water. All reactions were carried out in duplicate. PCR cycling conditions were as follows: one cycle of 45°C for 5 min and 95°C for 3 min followed by 39 cycles of 95°C for 10 s, 60.3°C for 10 s and 72°C for 30 s. A dissociation step of 65–95°C over 5 s was used for melt curve analysis for detection of non-specific products in the reaction. cDNA from each sample was pooled and diluted twofold, to serve as a standard curve and was included in each run to generate a standard curve for each primer set.

Bio Rad CFX Manager software was used to determine the cycle threshold of each sample (*C*_t_-Target) which was normalized to the geometric mean cycle threshold (*C*_t_) of the appropriate endogenous qPCR products (*C*_t_-Control) for the same sample [31–33]. Relative gene expression was calculated using the comparative *C*_t_ method (2-^ΔΔ^*C*_t_), where ΔΔ*C*_t_=(*C*_t. target_−*C*_t. reference_)_Time *x*_−(*C*_t. target_−*C*_t. reference_)_Time 0_, [34].

### 3.3 Insect cuticle preparation

*Aedes aegypti* (strain AeAe) were reared at room temperature (22°*C*±2°*C*) in distilled water to late (*L*_3−4_) instar and fed Tetramin fish food. *Tenebrio molitor* larvae were maintained at 27°C (±2°C) on wheat bran. *Aedes aegypti* larvae were removed from water and dried on Whatman No. 1 filter paper before flash freezing under liquid nitrogen. *Tenebrio molitor* larvae were washed in distilled water and flash frozen under liquid nitrogen before cutting the abdominal cuticle into 5×10 mm sections. Five mosquito and five *T. molitor* cuticles were subsequently fixed, with a small amount of expoxy resin adhesive, to a glass slide.

### 3.4 Spore probe preparation

*Metarhizium anisopliae* isolate ARSEF 4556 spores were harvested from cultures maintained on broken basmati rice [35]. Individual fungal spores were immobilized at the end of a V-shaped tipless AFM cantilever (Thermomicroscopes; electronic supplementary material, figure S1). Fungal spores were dusted onto a glass slide, previously cleaned with ethanol. Glue (Homebase glass glue) was applied to the AFM cantilever and a single conidium fixed to the AFM cantilever using the AFM itself [36–38]. To confirm attachment, the spore colloid probe was visualized under a VHX-600 Keyence microscope and later imaged with a scanning electron microscope.

### 3.5 Atomic force microscopy

**Figure 1.**
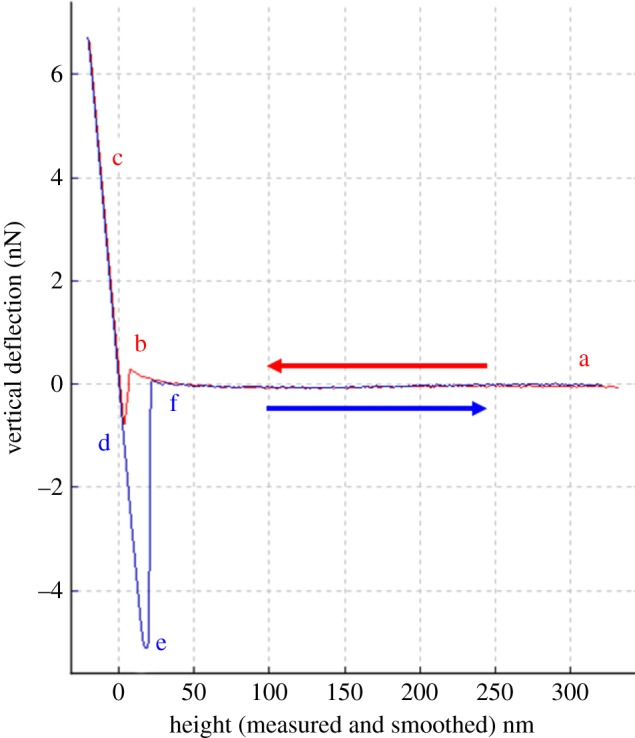
Typical force–distance curve showing a single adhesion event of *Metarhizium anisopliae* to glass. The red line is the approach curve and the blue line is the retraction curve. The cantilever is away from the sample surface (a), as the cantilever approaches the sample, initially the force is too small to provide a measureable deflection and remains in a neutral position at 0 nm, until reaching the contact point (b) where attractive forces (Van der Waals) overcome the cantilever spring constant and the tip jumps to contact with the sample. Once in contact the tip remains in contact as the separation between cantilever and sample decreases, causing a deflection of the cantilever (c). As the cantilever is retracted the tip remains in contact (d), owing to adhesion and the cantilever is deflected down (e). Eventually, the force on the cantilever is enough to overcome adhesion and the tip breaks free, returning to a neutral position (f).

A JPK nanowizard II AFM (JPK instruments, Berlin) was used to measure the adhesion forces between the spore probe and sample, by the vertical deflection of the cantilever. Cantilever deflection is measured as a change in the reflected laser path, resulting in a cantilever deflection–displacement curve (figure 1). Prior to force measurements, the cantilever was calibrated, to convert the deflection into a quantitative force measurement. The spring constant and sensitivity of cantilever were measured directly, by lowering the cantilever onto a clean glass surface submerged in deionized water, generating a spring constant of 0.26 Nm^−1^ and sensitivity of 53 nm V^−1^, these values were used in subsequent force calculations following Hooke's Law. Experiments were carried out in a fluid cell containing deionized water. Measurements were made on 121 and 115 different areas of the cuticle of *Tenebrio* and *Aedes*, respectively. Each measurement was replicated three to five times before moving to a new location on the cuticle. Measurements were recorded across five cuticles from *Aedes* and five from *Tenebrio*. All forces curves were normalized resulting in tip deflection of 0 nm where there was no interaction.

### 3.6 Epicuticular wax extraction

All insects were washed in deionized water and dried at 30°C (±2°C) prior to solvent extraction. Six replicates of five *Ae. aegypti* or two *T. molitor* larvae were washed in hexane for 10 s before discarding, the hexane fraction was subsequently dried under nitrogen gas before resuspending in 1 ml transesterification solution: HCl/MeOH (5% v/v) containing 0.01% butylated hydroxyltoluene (w/v). Samples were heated to 90°C for 90 min and allowed to cool to room temperature before incubating in 2 ml hexane with gentle agitation for 30 min. The hexane extract was collected and the sample extracted with hexane a further three times. All hexane fractions were pooled before drying under nitrogen gas. Heptadecanoic acid (C17:0) was added prior to extraction to act as an internal standard. Dried samples were stored at −80°C until required.

### 3.7 Gas chromatography-mass spectrometry

Prior to gas chromatography-mass spectrometry (GC-MS) analysis, samples were resuspended in hexane and 1 μl of each sample injected onto a Thermo TR-5 ms SQC gas chromatography (GC) column (15 m×0.25 mm×0.25 μm) (Themo Scientific, UK) which was eluted by a Trace GC Ultra (Thermo Scientific, UK). There was a 0.5 s pre-injection dwell time and a single wash with 1 μl chloroform followed by one pre-injection rinse. The temperature gradient for elution used an initial temperature of 50°C which was held for 1 min and then increased at 30°C per minute up to a final temperature of 300°C which was held for 5 min before returning to starting conditions. The eluent from the GC column was analysed by a DSQ II mass spectrometer which was used in positive ionization mode and a mass range for 33–650 amu in electron impact mode. An ion source temperature of 200°C, a multiplier voltage of 1215 V, 4.06 scans s^−1^, 2534 amu s^−1^ and a scan range of 33–600 amu were used. For compound identification in electron impact analysis, mass spectra from individual components were submitted to the NIST database within the Xcalibur software (Thermo Scientific, UK) and identifications confirmed by probability score and manual inspection of the experimental and theoretical data.

### 3.8 Statistical analysis

Comparison of adhesion force measurements between samples was analysed using a Kruskal–Wallis *H*-test. Pairwise comparisons were performed using Dunn's post-hoc with a Bonferroni correction for multiple comparisons. GC-MS data was analysed using a Mann–Whitney *U*-test and molecular datasets were analysed using two-way analysis of variance (ANOVA) with Tukey HSD post-test. Prior to analysis, gene expression data was subjected to a Box-Cox transformation, conforming to ANOVA assumption of homogeneity of variance [33]. All statistical analyses were carried out using MatLab R2013b and Rv. 3.0.2 [39].

## 4. Results

### 4.1 Gene expression

**Figure 2.**
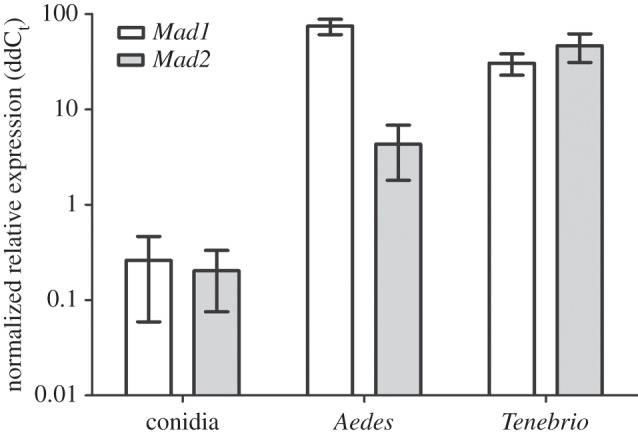
Upregulation of *Mad* genes indicative of a host–pathogen response. Expression of adhesin genes, 48 h post inoculation, analysed by qRT-PCR. *Mad1* genes are constitutively expressed in conidia, regardless of insect host. Data was presented as mean (±s.e.m.). Data normalized to average dC_t_ of conidia.

Adhesins (*Mad1* and *Mad2*) are fundamental in fungal adhesion and therefore pathogenicity. The genes for these components were analysed and shown to be constitutively expressed in conidia but upregulated in the presence of both *Aedes* and *Tenebrio* with no difference in expression of *Mad1* irrespective of insect host (figure 2; *F*_1,12_=7195, *p*=0.326). The only significant differences in expression between genes were recorded for *Aedes* (figure 2; *F*_2,12_=11.649, *p*=0.0199) with expression of *Mad2* being significantly lower in *Aedes* compared with the same genes expression in *Tenebrio* (figure 2; *F*_2,12_=49.792, *p*=0.0199).

### 4.2 Force measurements

Prior to force measurements the topography of a limited number of *Aedes* and *Tenebrio* cuticles was investigated using an AFM image scan (electronic supplementary material, figure S2). The surface of *Aedes* presented a rougher, more convoluted surface than *Tenebrio*, providing a greater surface area for attachment [40,41]. Contrary to previous research [42,43], the force curves generated were not reflective of a rougher surface topography.

**Figure 3.**
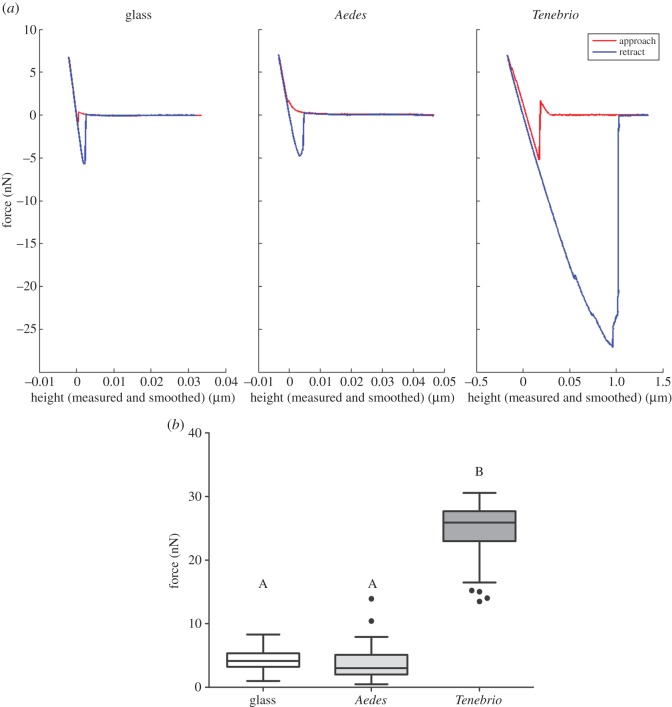
(*a*) *Metarhizium* conidia fail to adhere to *Aedes* cuticle. Typical force–distance curves observed for *Metarhizium* conidia to glass or *Aedes* and *Tenebrio* cuticle. Large force measurements, obtained in *Tenebrio*, indicative of a strong interaction are absent in glass and *Aedes* samples. (*b*) Average adhesion forces measured. Adhesion forces measured between *Metarhizium* conidia and *Aedes* (*n*=115), *Tenebrio* (*n*=121) and glass (*n*=100). Letters denote significant differences, Tukey whisker (25th and 75th percentile). Boxes denote interquartile range, bisected horizontally by median values; whiskers extend to 1.5×*interquartile* range beyond boxes; outliers are represented with black dots outside of whiskers.

The force interactions between the abdominal cuticle segments and an immobilized spore were determined using AFM in order to assess whether the elevated expression of the adhesion genes allowed for effective attachment of the spore to the cuticle, compared with non-attachment to an inert glass surface. Figure 3*a* shows typical force–distance curves, with a single adhesion event, of *M. anisopliae* to glass, *Aedes* and *Tenebrio*.

*Tenebrio* approach curves show a large jump to contact peak, indicative of strong attractive forces, occuring at a distance of approximately 0.05 μm (figure 3*a*). Typically, the cantilever snaps to the sample surface if the attractive force on the probe reaches or exceeds the value of the spring constant. In contrast to *Tenebrio*, force–distance curves measured between glass and *Aedes* show no notable attractive interactions within the approach curve (figure 3*a*).

Similar forces were measured between the negative control glass (*n*=100) and *Aedes* (*n*=115) (figure 2*a*). Forces generated on *Tenebrio* (*n*=121) cuticle were an order of magnitude greater than those of *Aedes* ($\chi_{2}^{2}=65.366$, *p*≤0.001). Indeed, the adhesion forces measured for *Tenebrio* were fivefold greater than the control sample, or *Aedes* (figure 3*b*; *Mdn*=25.855, *p*≤0.0001). There was no significant difference in forces measured between glass and *Aedes* (figure 3*b*; *Mdn*=4.1505, 2.9980, respectively, *p*=0.130) illustrating an absence of specific attachment to the cuticle of the mosquito.

Retraction curves demonstrated interesting differences; *Tenebrio* force–distance curves characteristically showed one adhesive peak over a large area with a sharp, de-adhesion to a neutral position, after a distance of approximately 1 μm. In contrast to this, retraction curves with multiple peaks and constant force plateaus at lower adhesion forces of approximately 0.5 nN were measured for *Aedes*, compared to weakest forces recorded in *Tenebrio* being between 15 and 20 nN. This indicates the possibility of other short-range molecular interactions, which are much weaker than those formed with *Tenebrio* (data not shown).

### 4.3 Epicuticular wax analysis

**Figure 4.**
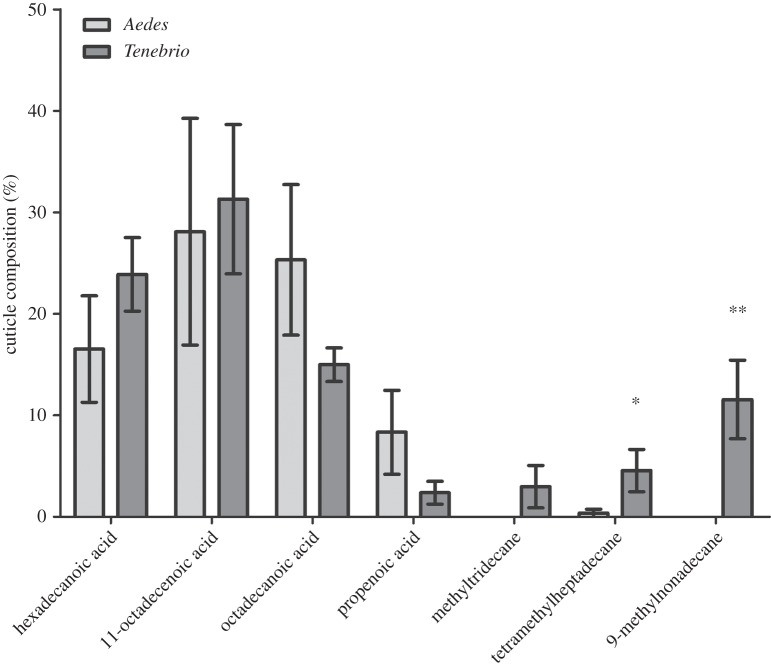
Long-chain fatty acids absent in mosquito larvae. Fatty acid composition in *Aedes* and *Tenebrio* larvae. Data presented as ±s.e.m. (*p≤0.05, ^**^p≤0.01).

In order to determine whether the chemical make-up of the cuticle itself may—at least partially—be a contributing factor with regard to successful attachment or not of the spore to the different tested insects, the majority lipid composition of cuticle was analysed by GC-MS analysis (electronic supplementary material, figure S3). Seven hydrocarbons were identified whose abundance was similar across the two insects (figure 4). The *Tenebrio* cuticle contained the long-chain hydrocarbons, tetramethylheptadecane and 9-methylnonadecane, whereas the *Aedes* cuticle did not (figure 4; *U*=23.00, *z*=2.263, *p*=0.031 and *U*=25.00, *z*=2.785, *p*=0.008, respectively).

## 5. Discussion

Adhesion of entomopathogenic fungi to host cuticle is considered to involve an initial binding of the conidia to the cuticle, largely through weaker hydrophobic forces, followed by consolidation and firm attachment [11,14].

This study found that there is an upregulation of *Mad* genes in response to exposure to *Aedes* cuticle which further corroborates work by Butt *et al.* [29] and is indicative of host–pathogen recognition. However, the inability of the fungus to establish firm attachment prevents the typical infection course from proceeding as the AFM data demonstrates that conidia of *M. anisopliae* fail to firmly adhere to the mosquito larvae cuticle, discrete force steps observed in both *Tenebrio* and *Aedes* force curves but absent in the glass force curves, may be attributed to the breaking force of a receptor–ligand pair [44]. While no firm attachment was determined for *Aedes*, the presence of weak short-range molecular interactions in *Aedes* further emphasizes recognition as suggested by the adhesion gene upregulation. Formation and strength of attachment is dependent upon the stability of molecular interactions, between the spore surface components and binding sites or receptors in the cuticle, under force [45], suggesting the initial recognition and binding to *Aedes* is extremely weak and not readily maintained. It has been shown that cells are able to regulate their receptor–ligand interactions, adapting them to the environments they are in [44].

Although preventing adhesion to the cuticle is a rare defence mechanism, it is well documented that hydrocarbon content of the waxy layer can influence fungal pathogenesis, with some compounds altering hydrophobicity or being fungistatic [12,46,47], in addition some insects actively secrete a variety of antimicrobial compounds on the cuticular surface. GC-MS analysis revealed no notable fungistatic compounds within the mosquito cuticle. There was, however, an absence of three long-chain hydrocarbons within the mosquito larval cuticle. For germination to occur, conidia require cuticular nutrients, namely long-chain fatty acids, lipids, sugars and amino acids [11,48], it is therefore feasible that the mosquito larval cuticle is not conducive for fungal development.

*Metarhizium* has demonstrated the ability to form firm attachment to a terrestrial host cuticle under water, indicating the ability of this fungus to adapt and form associations in an aquatic environment. In the mosquito larvae, the route of infection is more similar to the aquatic fungal pathogen *Culicinomyces clavisporus*, which is ingested rather than penetrating the cuticle [49], however, the upregulation of adhesin genes suggest there is recognition and an attempt to form attachment to the mosquito larvae. As a consequence, it can only be postulated that the cuticle itself is not providing an adequate substrate to further develop and, therefore, initiate full pathogenesis via traditional means.

## Data accessibility

Supporting data are deposited with dryad doi:10.5061/dryad.bg48n and in the electronic supplementary material.
